# Rapid versus conventional glucocorticoid tapering in pure membranous LN: a multicentre retrospective study

**DOI:** 10.1093/rap/rkag096

**Published:** 2026-08-07

**Authors:** Hidekazu Ikeuchi, Keiju Hiromura, Yoshiya Tanaka, Hiroko Sato, Koichiro Ohmura, Hiroki Hayashi, Kazuoto Hiramoto, Akiho Iwashita, Shinsuke Yasuda, Shinya Kaname, Yutaka Kawahito, Hiromi Shimada, Tomohiro Koga, Isao Matsumoto, Masahiro Ayano, Kazuro Kamada, Shoichi Maruyama, Tomonori Ishii, Satoshi Kubo, Shingo Nakayamada, Hayato Shimizu, Shuji Sumitomo, Yukio Yuzawa, Yuko Kaneko, Hironari Hanaoka, Akira Onishi, Akio Morinobu, Tadashi Hosoya, Taiki Yamaguchi, Takahisa Kawakami, Aki Sakashita, Takahiro Seno, Takashi Kida, Hiroaki Dobashi, Takuya Tomokawa, Atsushi Kawakami, Yuya Kondo, Hiroaki Niiro, Michihito Kono, Sawako Kato, Kayaho Maeda, Tatsuya Atsumi

**Affiliations:** Department of Nephrology and Rheumatology, Gunma University Graduate School of Medicine, Maebashi, Japan; Department of Nephrology and Rheumatology, Gunma University Graduate School of Medicine, Maebashi, Japan; Department of Molecular Targeted Therapeutics, University of Occupational and Environmental Health, Kitakyushu, Japan; Department of Rheumatology, Tohoku University Hospital, Sendai, Japan; Department of Rheumatology, Kobe City Medical Center General Hospital, Kobe, Japan; Department of Nephrology, Fujita Health University School of Medicine, Toyoake, Japan; Division of Rheumatology, Department of Internal Medicine, Keio University School of Medicine, Tokyo, Japan; Department of Rheumatology and Clinical Immunology, Kyoto University Graduate School of Medicine, Kyoto, Japan; Department of Rheumatology, Institute of Science Tokyo, Tokyo, Japan; Department of Nephrology and Rheumatology, Kyorin University School of Medicine, Mitaka, Japan; Department of Internal Medicine, Kichijoji Asahi Hospital, Tokyo, Japan; Inflammation and Immunology, Kyoto Prefectural University of Medicine, Kyoto, Japan; Department of Hematology, Rheumatology and Respiratory Medicine, Kagawa University, Kagawa, Japan; Department of Immunology and Rheumatology, Division of Advanced Preventive Medical Sciences, Nagasaki University Graduate School of Biomedical Sciences, Nagasaki, Japan; Department of Rheumatology, Institute of Medicine, University of Tsukuba, Tsukuba, Japan; Department of Medicine and Biosystemic Science, Graduate School of Medical Sciences, Kyushu University, Fukuoka, Japan; Department of Rheumatology, Endocrinology and Nephrology, Faculty of Medicine and Graduate School of Medicine, Hokkaido University, Sapporo, Japan; Department of Nephrology, Nagoya University Graduate School of Medicine, Nagoya, Japan; Department of Rheumatology, Tohoku University Hospital, Sendai, Japan; Department of Hematology and Rheumatology, Tohoku Medical and Pharmaceutical University, Sendai, Japan; The First Department of Internal Medicine, University of Occupational and Environmental Health, Kitakyushu, Japan; The First Department of Internal Medicine, University of Occupational and Environmental Health, Kitakyushu, Japan; Department of Rheumatology, Kobe City Medical Center General Hospital, Kobe, Japan; Department of General Internal Medicine, Kobe City Medical Center General Hospital, Kobe, Japan; Department of Rheumatology, Kobe City Medical Center General Hospital, Kobe, Japan; Department of Nephrology, Fujita Health University School of Medicine, Toyoake, Japan; Division of Rheumatology, Department of Internal Medicine, Keio University School of Medicine, Tokyo, Japan; Division of Rheumatology, Department of Internal Medicine, Keio University School of Medicine, Tokyo, Japan; Rheumatology and Clinical Immunology, Saitama Medical Center, Saitama Medical University, Kawagoe, Japan; Department of Advanced Medicine for Rheumatic Diseases, Kyoto University Graduate School of Medicine, Kyoto, Japan; Department of Rheumatology and Clinical Immunology, Kyoto University Graduate School of Medicine, Kyoto, Japan; Department of Rheumatology, Institute of Science Tokyo, Tokyo, Japan; Department of Rheumatology, Institute of Science Tokyo, Tokyo, Japan; Department of Nephrology and Rheumatology, Kyorin University School of Medicine, Mitaka, Japan; Inflammation and Immunology, Kyoto Prefectural University of Medicine, Kyoto, Japan; Inflammation and Immunology, Kyoto Prefectural University of Medicine, Kyoto, Japan; Inflammation and Immunology, Kyoto Prefectural University of Medicine, Kyoto, Japan; Department of Hematology, Rheumatology and Respiratory Medicine, Kagawa University, Kagawa, Japan; Department of Immunology and Rheumatology, Division of Advanced Preventive Medical Sciences, Nagasaki University Graduate School of Biomedical Sciences, Nagasaki, Japan; DEJIMA Infectious Disease Research Alliance (DIDA), Nagasaki University, Nagasaki, Japan; Clinical Research Center, Nagasaki University Hospital, Nagasaki, Japan; Research Career Support Office, Faculty of Biomedical Sciences, Nagasaki University, Nagasaki, Japan; Department of Rheumatology, Institute of Medicine, University of Tsukuba, Tsukuba, Japan; Department of Medical Education, Graduate School of Medical Sciences, Kyushu University, Fukuoka, Japan; Department of Rheumatology, Endocrinology and Nephrology, Faculty of Medicine and Graduate School of Medicine, Hokkaido University, Sapporo, Japan; Department of Nephrology, Nagoya University Graduate School of Medicine, Nagoya, Japan; Department of Nephrology, Nagoya University Graduate School of Medicine, Nagoya, Japan; Department of Rheumatology, Endocrinology and Nephrology, Faculty of Medicine and Graduate School of Medicine, Hokkaido University, Sapporo, Japan

**Keywords:** SLE and autoimmunity, renal, histopathology, biological therapies, immunosuppressants, immunotherapy, outcome measures

## Abstract

**Objectives:**

While recent guidelines recommend early glucocorticoid (GC) tapering for LN, supporting evidence for pure membranous LN remains limited. The present study investigated the impact of rapid GC tapering on renal outcomes in this population.

**Methods:**

We performed a multicentre retrospective study across 16 centres in Japan, including 67 patients with biopsy-proven pure membranous LN who initiated GC therapy between 2003 and 2023. Patients were classified into rapid tapering (GC dose ≤7.5 mg/day at 6 months) and conventional tapering (>7.5 mg/day at 6 months) groups. The primary endpoint was the partial renal response (PRR) at 12 months. Secondary endpoints included the complete renal response (CRR), relapse and severe adverse events (SAEs). Adjusted risk ratios (aRRs) were estimated using modified Poisson regression models for failure to achieve PRR/CRR.

**Results:**

Fifteen patients underwent rapid tapering and 52 conventional tapering. Baseline characteristics were generally comparable. At 12 months, PRR was achieved in 85.7% of the rapid group and 84.3% of the conventional group (aRR for failure 1.04; 95% CI 0.17–6.39). CRR rates at 12 months were 64.3% and 56.9%, respectively (aRR for failure 0.90; 95% CI 0.40–2.02). At 24 months, PRR and CRR did not differ statistically between groups. Relapse occurred in 20.0% versus 7.7% (*P* = 0.185). No SAEs were reported in the rapid group, while three occurred in the conventional group.

**Conclusions:**

Rapid GC tapering to ≤7.5 mg/day at 6 months appeared feasible in a subset of patients with pure membranous LN, but the findings are preliminary and should be interpreted cautiously because of the small rapid-taper group and imprecise estimates.

## Graphical abstract

**Graphical Abstract:**
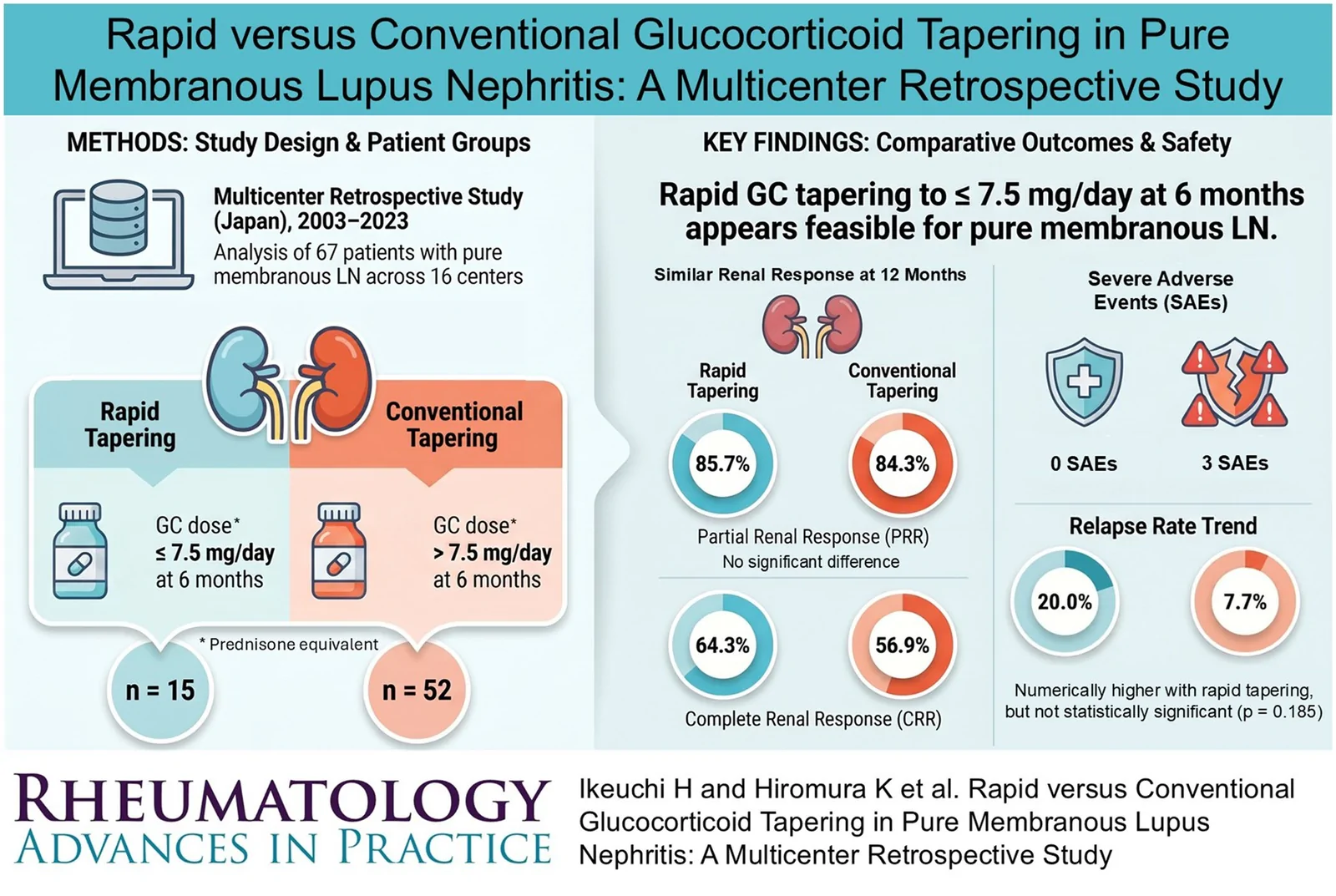

Key messages

- Rapid glucocorticoid tapering appeared feasible in a subset of patients with pure membranous LN.
- Renal response rates did not differ statistically from conventional tapering, but effect estimates were imprecise.
- Prospective studies should confirm optimal glucocorticoid tapering strategies in this population.

## Introduction

LN is one of the most severe clinical manifestations of SLE. If not properly managed, it represents a major cause of morbidity and mortality and may progress to end-stage kidney disease [1]. Glucocorticoid (GC) therapy has historically improved survival and reduced the risk of kidney failure [2]; however, its long-term use results in cumulative toxicity and irreversible organ damage, ultimately impairing quality of life [3–5]. Consequently, minimizing GC exposure has become a central therapeutic goal, and the introduction of immunosuppressive agents, such as cyclophosphamide and MMF, has made dose reductions achievable [6–8].

Recent guidelines have further reinforced this strategy. The 2014 treat-to-target (T2T) recommendations for SLE emphasized reducing GC to the lowest possible dose required for disease control and advocated complete withdrawal if feasible [9]. Similarly, the 2019 recommendations of the European Alliance of Associations for Rheumatology and the European Renal Association–European Dialysis and Transplant Association (EULAR/ERA-EDTA) advised tapering prednisone to ≤7.5 mg/day within 3–6 months for patients with proliferative LN (Class III/IV) [10].

To assess the impact of early GC tapering on the renal outcomes of proliferative LN, we conducted a multicentre retrospective study in Japan. Referring to the 2019 EULAR/ERA-EDTA recommendations [10], we compared a rapid tapering group, in which the GC dose was reduced to ≤7.5 mg/day (prednisone equivalent) 6 months after treatment initiation, with a conventional tapering group, in which the dose remained >7.5 mg/day at 6 months. The study demonstrated that treatment responses at 12 months, as well as relapse rates and the incidence of severe adverse events (SAEs) over 2 years, were similar between the two groups [11].

In contrast, pure membranous LN has a distinct pathophysiology, and evidence regarding optimal GC management remains limited [12]. It remains unclear whether rapid tapering may be safely applied to pure membranous LN. Therefore, building upon our findings on proliferative LN, we conducted a multicentre retrospective study to evaluate the efficacy and safety of rapid GC tapering for pure membranous LN.

## Methods

### Study design and setting

We conducted a multicentre, retrospective observational study across 16 centres in Japan. Data were collected from the medical records of LN patients who initiated GC treatment between January 2003 and August 2023 and had previously undergone renal biopsy.

### Patients

Patients were eligible if they had a diagnosis of SLE according to the 1997 ACR criteria or the 2019 EULAR/ACR classification criteria [13, 14] and had developed LN. All patients underwent renal biopsy and were classified as having biopsy-confirmed pure membranous LN (Class V) according to the International Society of Nephrology/Renal Pathology Society (ISN/RPS) classification [15]. Eligible patients were required to have received GC therapy, with quantitative urine protein and serum creatinine measurements available both before the initiation of and after 1 year of GC therapy. Patients who underwent renal biopsy within 3 months after starting GC therapy were also included. Only patients receiving initial remission induction as part of the LN treatment were enrolled, and no age restrictions were applied. Patients were excluded if they had received GC or other immunosuppressive therapy (excluding HCQ) for more than 3 months prior to renal biopsy.

### Data collection and variables

The following clinical data were collected: age at treatment initiation, sex, weight, renal biopsy classification, initial GC dose, GC dose at 6 months, the use of pulse intravenous methylprednisolone (pulse IV MP), concomitant immunosuppressants (within 2 years after GC initiation) and concomitant HCQ (within 2 years). GC dose refers to the daily oral prednisone equivalent dose. Laboratory data included serum creatinine, the estimated glomerular filtration rate (eGFR; assessed within 2 years after GC initiation and at the last observation), daily urinary protein excretion before GC initiation (expressed as the urine protein-to-creatinine ratio [UPCR, g/Cr]) and during the follow-up (within 2 years and at the last observation), the anti-dsDNA antibody titer (within 2 years) and baseline complement levels (C3, C4; within 2 years). Additional variables included the date of the last observation, the relapse status (within 2 years) and the presence and type of SAEs. SAEs were defined as grade ≥3 according to the Common Terminology Criteria for Adverse Events, version 5.0. Regarding values below the detection limit, CH50 was set to 4, C3 to 17.3, C4 to 1 and the anti-dsDNA antibody to 1. Antibody levels above the upper detection limit were set to 500.

### Definition of GC tapering groups and outcomes

The main exposure was the speed of the GC reduction. Patients were classified into two groups according to their tapering strategy based on the 2019 EULAR/ERA-EDTA recommendations [10]. The rapid tapering group was defined as patients receiving a GC dose ≤7.5 mg/day 6 months after treatment initiation, whereas the conventional tapering group included those receiving a GC dose >7.5 mg/day at 6 months.

The primary endpoint was the partial renal response (PRR) rate at 12 months. Secondary endpoints included the PRR rate at 24 months and the complete renal response (CRR) rate at 12 and 24 months. PRR and CRR were defined according to the BLISS-LN trial [16]. Briefly, PRR was defined as UPCR ≤0.7 g/gCr (or ≤70 mg/mmol) and either eGFR ≥60 ml/min/1.73 m^2^ or no decline ≥20% from baseline. CRR was defined as UPCR <0.5 g/gCr (or <50 mg/mmol) and either eGFR ≥90 ml/min/1.73 m^2^ or no decline ≥10% from baseline, both without rescue therapy. Relapse was defined as an increase in GC dose ≥10 mg/day for at least 1 month due to disease activity.

### Statistical analysis

Patient characteristics and clinical outcomes are summarized according to GC tapering groups. Categorical variables are presented as numbers and percentages, and continuous variables as means with SDs or medians with interquartile ranges, as appropriate. In statistical comparisons, Fisher’s exact test was applied to categorical variables and the Mann–Whitney U test was applied to continuous variables. Relapse-free survival after the initiation of GC therapy was estimated using the Kaplan–Meier method, with between-group comparisons performed using the log-rank test.

Since the incidence of failure to achieve PRR or CRR at 12 or 24 months was expected to exceed 10%, modified Poisson regression models with robust error variance were used to estimate adjusted risk ratios (aRRs) and 95% CIs for failure to achieve PRR/CRR in the rapid GC reduction group relative to the conventional group. Covariates included in the regression model were selected based on clinical relevance and potential confounders: age, initial UPCR, initial eGFR, initial GC dose, use of pulse IV MP, use of strong immunosuppressants (MMF, cyclophosphamide or rituximab), use of HCQ during the remission induction phase (0–6 months) and the year of GC initiation (categorized as before or after the end of 2015, when the treatment strategy markedly changed in Japan because the T2T strategy [9] for SLE was published in 2014 and MMF received insurance approval in 2016). Two-tailed *P*-values were obtained using the Wald test, with *P* < 0.05 being significant. All analyses were conducted using R software version 4.4.1 (R Foundation for Statistical Computing, Vienna, Austria).

### Ethics approval

The present study was conducted in accordance with the Declaration of Helsinki. The protocol was approved by the Institutional Review Board (IRB) of Kobe City Medical Center General Hospital (reference number: 23126) and then by the Ethics Committees or IRBs of all participating institutions, including the IRB of Gunma University Hospital (reference number: 2023–075). Due to its retrospective design and the use of anonymized data, the need for individual informed consent was waived. An opt-out approach was instead adopted, whereby study information was disclosed and patients were given the opportunity to decline participation.

## Results

### Patient clinical background and treatment

Sixty-seven patients with biopsy-proven pure membranous LN (ISN/RPS Class V) were included in the analysis. Of these, 15 patients were classified as rapid GC reducers (GC dose ≤7.5 mg/day at 6 months) and 52 as conventional GC reducers (GC equivalent dose >7.5 mg/day at 6 months).

**Figure 1:**
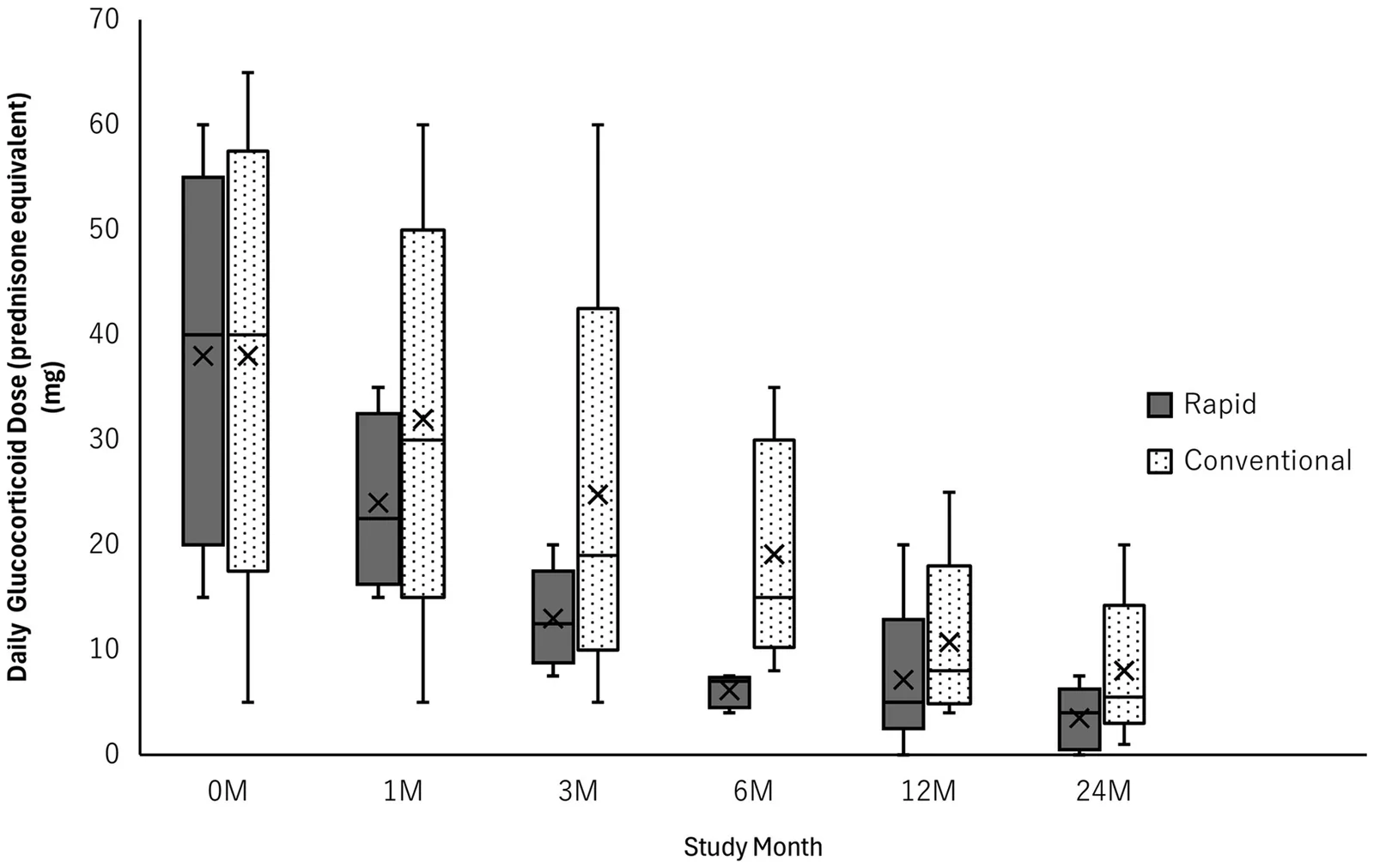
Daily oral glucocorticoid (GC) doses over time following treatment initiation. The initial oral GC dose (prednisone equivalent, mg/day) and subsequent doses at 1, 3, 6, 12 and 24 months are displayed. Data are represented as the mean (×), median (horizontal line) and interquartile ranges (box and whisker plots) of the oral GC dosage at each time point. The rapid GC reducer group (prednisone equivalent ≤7.5 mg/day at 6 months) is shown in grey, while the conventional GC reducer group (prednisone equivalent >7.5 mg/day at 6 months) is shown with dotted spots

The baseline characteristics of the two groups are shown in Table 1. No statistically significant differences were observed in demographics, clinical parameters (UPCR, eGFR, creatinine, SLEDAI 2000, anti-dsDNA antibodies and complement levels) between the groups. The treatment variables of the two groups are shown in Table 2. Similarly, no statistically significant differences were observed in initial GC dose, the frequency of pulse IV MP, use and distribution of immunosuppressants and HCQ use during the first 6 months between the groups. The proportion of patients who initiated GC therapy after 2015 was higher in the rapid-taper group than in the conventional-taper group [11 (73.3%) vs. 24 (46.2%)], although the difference did not reach statistical significance (*P* = 0.082). Figure 1 shows the trajectory of daily oral GC doses in the rapid and conventional reduction groups across the 24-month follow-up period, demonstrating a faster decline in the rapid reduction group.

**Table 1:** Patient characteristics.

	Rapid (*n* = 15)	Conventional (*n* = 52)	*P*-value
Female sex—no. (%)	11 (73.3)	45 (86.5)	0.248
Age—years	45.7 (17.5)	39.3 (13.3)	0.131
Body weight—kg	57.1 (12.2)	54.3 (9.6)	0.520
Urine protein-to-creatinine ratio	2.4 (2.5)	3.3 (2.8)	0.288
eGFR—ml/min/1.73 m^2^	96.2 (30.2)	102.4 (27.8)	0.447
Creatinine—mg/dl	0.62 (0.13)	0.60 (0.27)	0.807
SLEDAI-2K	15.0 (4.94)	14.4 (7.22)	0.766
Biomarkers			
Anti-dsDNA antibodies—U/ml	95.1 (201.3)	40.4 (115.9)	0.193
Complement C3—mg/dl	64.5 (29.1)	73.2 (32.7)	0.359
Complement C4—mg/dl	13.0 (9.5)	12.8 (8.5)	0.943

Data are presented as no. (%) or mean (SD).

eGFR: estimated glomerular filtration rate; SLEDAI-2K: SLEDAI 2000.

**Table 2:** Patient treatments.

	Rapid (*n* = 15)	Conventional (*n* = 52)	*P*-value
Initial GC dose—mg/kg/day	0.70 (0.30)	0.78 (0.25)	0.296
pulse IV MP—no. (%)	5 (33.3)	7 (13.5)	0.121
Strong IS^a^ use during 0–6 months—no. (%)	9 (60.0)	26 (50.0)	0.566
IVCY—no. (%)	1 (6.7)	3 (5.8)	>0.999
MMF—no. (%)	8 (53.3)	23 (44.2)	0.569
BEL—no. (%)	0 (0.0)	2 (3.8)	>0.999
RTX—no. (%)	1 (6.7)	0 (0.0)	0.224
CNIs—no. (%)	4 (26.7)	15 (28.8)	>0.999
Combination therapy—no. (%)	2 (13.3)	4 (7.7)	0.609
HCQ use during 0–6 months—no. (%)	9 (60.0)	19 (36.5)	0.140

Combination therapy consisted of MMF combined with either BEL or CNIs. Data are presented as no. (%) or mean (SD).

a Strong IS indicates cyclophosphamide, MMF or rituximab.

GC: glucocorticoid; IV MP: intravenous methylprednisolone; IS: immunosuppressant; IVCY: intravenous cyclophosphamide; BEL: belimumab; RTX: rituximab; CNIs: calcineurin inhibitors, including tacrolimus or ciclosporin A.

### Renal outcomes after two GC reduction protocols

The primary outcome, the achievement of PRR at 12 months, was observed in 12/14 (85.7%) patients in the rapid reduction group and 43/51 (84.3%) in the conventional group (Table 3). After adjustments for age, baseline UPCR, eGFR, initial GC dose, pulse IV MP use, use of immunosuppressants and HCQ use, there was no significant difference in the risk of failing to achieve PRR between the groups (aRR for failure 1.04; 95% CI 0.17–6.39; *P* = 0.963).

**Table 3:** Primary and major secondary endpoints.

	Rapid	Conventional	aRR	95% CI	*P*-value
Primary endpoint					
PRR at 12 months—no. (%)	12/14 (85.7)	43/51 (84.3)	1.04	0.17, 6.39	0.963
Secondary endpoints					
CRR at 12 months—no. (%)	9/14 (64.3)	29/51 (56.9)	0.90	0.40, 2.02	0.791
PRR at 24 months—no. (%)	10/12 (83.3)	37/46 (80.4)	0.70	0.09, 5.20	0.727
CRR at 24 months—no. (%)	6/12 (50.0)	21/46 (45.7)	1.11	0.51, 2.41	0.801

PRR: partial renal response; CRR: complete renal response; aRR: adjusted risk ratio for failure to achieve the stated renal outcome.

Regarding secondary outcomes, CRR at 12 months was achieved in 9/14 (64.3%) patients in the rapid group and 29/51 (56.9%) in the conventional group, with no significant difference after adjustments (aRR for failure 0.90; 95% CI 0.40–2.02; *P* = 0.791). At 24 months, PRR was achieved in 10/12 (83.3%) and 37/46 (80.4%) patients, respectively, and CRR in 6/12 (50.0%) and 21/46 (45.7%) patients. Adjusted analyses again showed no significant differences between the groups (PRR: aRR for failure 0.70; 95% CI 0.09–5.20; *P* = 0.727; CRR: aRR for failure 1.11; 95% CI 0.51–2.41; *P* = 0.801). Renal response data were unavailable for one patient at 12 months and three patients at 24 months in the rapid-taper group, and for one patient at 12 months and six patients at 24 months in the conventional-taper group. These patients were excluded from the response analyses at the corresponding time points, and no imputation was performed.

Regarding renal function, the changes in eGFR from baseline to 24 months did not differ significantly between the two groups. The mean change in eGFR at 24 months was −8.97 (11.64) ml/min/1.73 m² in the rapid reduction group and −16.39 (15.51) ml/min/1.73 m² in the conventional group (*P* = 0.129).

### Relapse and SAEs

**Figure 2:**
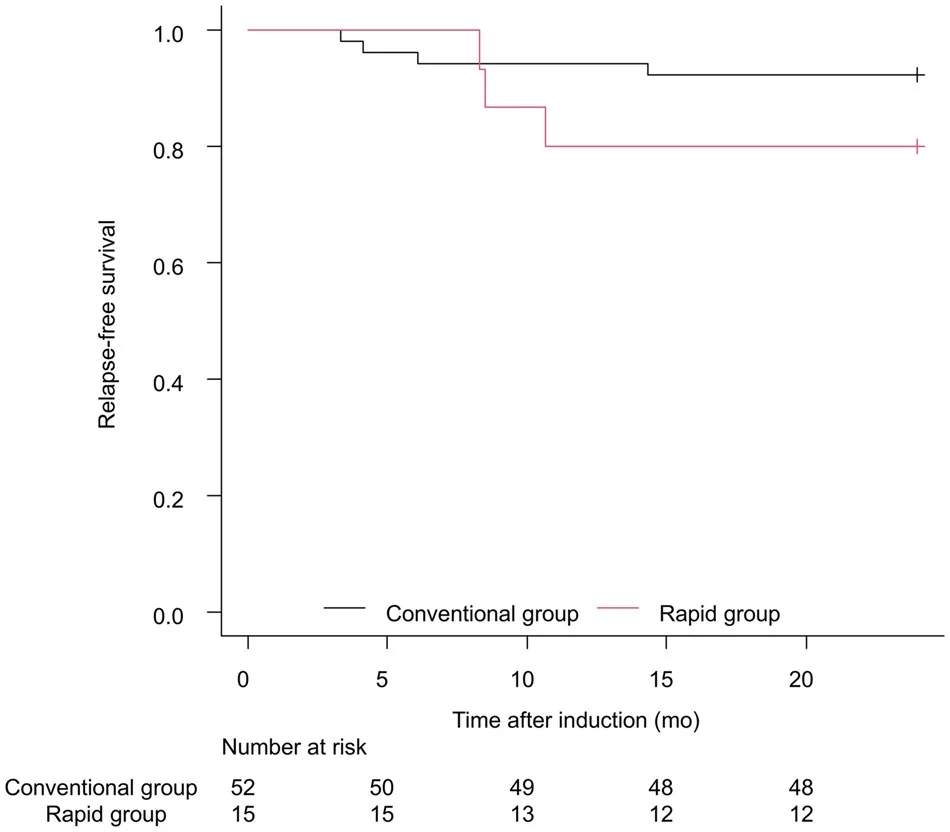
Relapse-free rates over 2 years for rapid and conventional GC reducers. Kaplan–Meier curves showing relapse-free rates over 2 years for conventional GC reducers (black line) and rapid GC reducers (red line) following GC treatment initiation

Relapse-free survival rates over the 2-year period are presented in Fig. 2. Relapse was observed in 3/15 (20.0%) patients in the rapid GC reduction group and 4/52 (7.7%) patients in the conventional group. The Kaplan–Meier analysis showed no significant difference in relapse-free survival between the two groups (log-rank, *P* = 0.185).

No SAEs were reported in the rapid GC reduction group (0/15), whereas SAEs occurred in three patients in the conventional group (3/52) (Table 4), including one case each of leukocytopenia, herpes zoster and hyponatraemia. The difference in the incidence of SAEs between the groups was not statistically significant (*P* > 0.999).

**Table 4:** Serious adverse events during the first 24 months.

	Rapid (*n* = 15)	Conventional (*n* = 52)	*P*-value
Patients with ≥1 SAE—no. (%)	0 (0)	3 (5.8)	>0.999
Leukocytopenia	0 (0)	1 (1.9)	
Herpes zoster	0 (0)	1 (1.9)	
Hyponatremia	0 (0)	1 (1.9)	

SAEs were defined as grade ≥3 according to the Common Terminology Criteria for Adverse Events, version 5.0. Data are presented as no. (%).

SAE: serious adverse event.

## Discussion

In this multicentre retrospective cohort of patients with biopsy-confirmed pure membranous LN, patients who achieved rapid tapering of GC to ≤7.5 mg/day at 6 months showed renal response rates that were not statistically worse than those observed with conventional tapering. Relapse and SAE rates also did not differ statistically between the groups. These findings provide preliminary real-world support for the feasibility of early GC minimization in patients with pure membranous LN who were able to undergo rapid tapering in clinical practice. However, because the rapid-taper group was small and defined according to a post-baseline treatment course, the results should not be interpreted as evidence of equivalence, non-inferiority, or causal evidence of safety.

Pure membranous LN is uncommon, accounting for approximately 10%–20% of all LN cases [17–19], and typically presents with nephrotic-range proteinuria [18]. Although historically considered to have a more favourable renal prognosis than proliferative LN [18, 20, 21], more recent studies have challenged this perception, reporting outcomes that are not necessarily superior [22]. In another multicentre registry study conducted in Japan, no significant differences in renal or patient survival were observed between pure membranous LN and proliferative LN during a median follow-up of 5 years [23]. One possible explanation is the lower intensity of initial therapy, with the reduced use of immunosuppressants and lower GC exposure [22, 23], highlighting the importance of selecting optimal GC regimens for pure membranous LN.

Several international guidelines and recommendations have addressed the treatment of LN; however, most evidence derives from studies of proliferative LN, and Class V-specific evidence remains limited [10, 24–26]. In the 2019 EULAR/ERA-EDTA recommendations, a specific regimen was proposed for pure Class V LN with nephrotic-range proteinuria or persistent proteinuria despite renin–angiotensin system blockade, consisting of MMF plus pulse IV MP followed by oral GC starting at 20 mg/day and tapered to ≤5 mg/day within 3 months [10]. The 2025 EULAR update represents a shift towards a unified treatment framework for active LN, recommending pulse IV MP followed by oral GC tapering to ≤5 mg/day by 4–6 months and early combination therapy with MMF or cyclophosphamide-based regimens plus belimumab, a calcineurin inhibitor, or obinutuzumab, particularly in patients with poor prognostic factors [24]. Notably, the recommendation for initial immune therapy no longer distinguishes between proliferative and membranous LN, although the update acknowledges that pure Class V LN remains underrepresented in clinical trials and that less intensive GC regimens may be appropriate for Class V LN with subnephrotic-range proteinuria [24]. Similarly, the 2024 KDIGO guideline presented pure membranous LN mainly as a Practice Point, suggesting GC plus immunosuppressive therapy with consideration of moderate or reduced-dose GC regimens [25], whereas the 2024 ACR guideline conditionally recommended GC, MMF and a calcineurin inhibitor for pure Class V LN with clinically relevant proteinuria [26]. Thus, although recent guidelines consistently emphasize GC minimization and background immunosuppression, Class V-specific evidence remains limited, and the optimal GC tapering strategy for pure membranous LN has not been established.

In the present study, rapid tapering was defined as achieving a GC dose ≤7.5 mg/day at 6 months after treatment initiation. This definition was consistent with that employed in our previous investigation of rapid tapering in proliferative LN within the same cohort [11]. As noted above, the 2025 EULAR update advocates a more ambitious approach, namely, tapering oral GC to ≤5 mg/day by 4–6 months, facilitated by the early use of potent combination therapies [24]. However, in our cohort, only 6 of 67 patients (9.0%) received these modern combination regimens, and only a single patient (1.5%) met the ≤5 mg/day criterion at 6 months. Even when applying the 6-month threshold of prednisone equivalent ≤7.5 mg/day, only 15 of 67 patients (22.4%) achieved rapid tapering. Although cases from earlier treatment periods were included, these findings suggest that rapid tapering remains uncommon in Japan, reflecting a gap between evolving international recommendations and real-world historical practice.

This study has several limitations. First, the exposure was defined by the GC dose at 6 months, which is a post-baseline variable. Patients with an early favourable response, fewer extrarenal manifestations, better tolerability or treatment by physicians favouring steroid minimization may have been more likely to be classified into the rapid-taper group, creating a risk of reverse causation and confounding by indication. A descriptive post-hoc comparison showed that a ≥ 50% reduction in proteinuria at 3 months was observed in 27/47 patients (57.4%) in the conventional-taper group and 9/12 patients (75.0%) in the rapid-taper group (Fisher’s exact test, *P* = 0.334); however, this analysis was underpowered and cannot exclude bias. Second, despite multivariable adjustment, residual confounding is likely because membranous-specific factors, including nephrotic-syndrome severity, histologic activity/chronicity, antiphospholipid status and detailed immunosuppressive dosing and duration, were not fully captured. In addition, although not statistically significant, clinically relevant imbalances, including numerically lower baseline proteinuria, more frequent pulse IV MP, greater HCQ use and a higher proportion of patients treated after 2015 in the rapid-taper group, may have favoured outcomes in that group. Third, the rapid-taper group was small and event numbers were limited, leading to imprecise estimates. Although relapse-free survival did not differ statistically between groups, relapse occurred numerically more frequently in the rapid-taper group (20.0% vs. 7.7%). Given this small sample size, the study was underpowered to determine whether this represents a true difference, and clinically important differences in relapse or adverse outcomes cannot be excluded. Fourth, safety analyses were limited to grade ≥3 SAEs and did not capture overall GC-related toxicity. Non-serious infections, other milder but clinically relevant steroid-related complications and serial assessments using the SLICC/ACR Damage Index were not systematically collected, precluding evaluation of overall treatment-related damage accrual. Finally, because recent guidelines increasingly recommend lower initial GC doses and early combination therapy, the applicability of these findings to contemporary treatment strategies requires further evaluation.

## Conclusion

In conclusion, in this small multicentre retrospective cohort of pure membranous LN, patients who achieved GC tapering to ≤7.5 mg/day at 6 months had renal response rates that did not differ statistically from those in patients managed with conventional tapering. These findings provide preliminary, hypothesis-generating real-world evidence supporting the feasibility of early GC minimization in this population. However, because the rapid-taper group was small and exposure was defined post-baseline, the results should not be interpreted as evidence of equivalence, non-inferiority or causal safety. Prospective studies are needed to identify patients with pure membranous LN who can safely undergo early GC minimization.

## Data Availability

Data are available upon reasonable request.
